# American Heart Association High Blood Pressure Protocol 2017: A Literature Review

**DOI:** 10.7759/cureus.3230

**Published:** 2018-08-29

**Authors:** Asad Ali, Muhammad Abu Zar, Ahmad Kamal, Amber E Faquih, Chandur Bhan, Waleed Iftikhar, Muhammad Bilal Malik, Malik Qistas Ahmad, Nouman Safdar Ali, Shahzad Ahmed Sami, FNU Jitidhar, Abbas M Cheema, Annum Zulfiqar

**Affiliations:** 1 Medicine, CMH Lahore Medical College and Institute of Dentistry, Lahore, PAK; 2 Hematology and Oncology, The University of Arizona, Tucson, USA; 3 Graduate, Dow University of Health Sciences, Karachi, PAK; 4 Internal Medicine, Chandka Medical College Hospital, Larkana, PAK; 5 Internal Medicine, CMH Lahore Medical College and Institute of Dentistry, Lahore, PAK; 6 Internal Medicine, Shifa College of Medicine, Islamabad, PAK; 7 Hematology-Oncology, University of Arizona Cancer Center, Tucson, USA; 8 Medicine, Jinnah Hospital/Allama Iqbal Medical College, Lahore, PAK; 9 Internal Medicine, Orthopedic and Medical Institute, Karachi, PAK; 10 Internal Medicine, Combined Military Hospital, Lahore, PAK; 11 Internal Medicine, Sheikh Zayed Medical College/Hospital, Lahore, PAK

**Keywords:** cardiovascular events, pharmacologic treatment, reduced ejection fraction, comorbidities, peripheral artery disease, chronic kidney disease (ckd), salt-sensitive, anti-hypertensive, intra cranial, atrial fibrillation

## Abstract

Hypertension is the most prevalent clinical symptom arising from various cardiovascular disorders. Likewise, it is considered a precursor or sequelae to the development of acute coronary artery disease and congestive heath failure (CHF). Hypertension has been considered a cardinal criterion to determine cardiovascular function. According to the World Health Organization (WHO) global observatory data, hypertension causes more than 7.5 million deaths a year, about 12.8% of the total human mortality. Similarly, the Center for Disease Control (CDC) states that 35% of the American adults have been estimated to have a persistently high blood pressure, which makes it about one in every three adults. Hypertension is a modifiable symptom that can be managed through pharmacological and non-pharmacological methods and standard protocols set forth by the American Heart Association (AHA). With new findings from various clinical trials related to the management of hypertension, new developments and recommendations have been made to update the previously established protocols for hypertension. This article aims to discuss and dissect the modern updates of hypertension management as comprehensively elaborated in the 2017 Hypertension Clinical Practice Guidelines.

## Introduction and background

Hypertension (HTN) is part of a clinical syndrome that results from multifaceted etiologies and can contribute to the development of complex cardiovascular disorders. In addition, it is a significant clinical symptom that directly relates to a dysfunctional cardiovascular system. HTN can be of two types: essential or secondary. In essential HTN, the etiology is unknown while in secondary HTN, a known pathological disorder is the cause. Hypertension (HTN) is primarily observed when there are pressure changes in the vascular system secondary to an abnormal cardiovascular fluid regulation and/or loss of vascular/arterial tone. It can also be due to abnormal contractility and/or aberrant electrical conductivity of the heart. Moreover, HTN can also be caused by conditions that are not organ-based and that predispose an individual to have these pressure changes.

The new definition of hypertension

HTN is diagnosed through non-invasive means with the application of a sphygmomanometer. It is necessary to ensure that blood pressure (BP) measurements are undertaken accurately. The apparatus determines the systolic blood pressure (SBP) and diastolic blood pressure (DBP) of an individual in millimeters of mercury (mm of Hg). According to the updated guideline [1] provided by the American Heart Association (AHA), a new approach for the classification of blood BP in adults is recommended. The new guideline recommends the use of an average SBP ≥130 mm Hg or an average DBP ≥80 mm Hg instead of the values from recent previous guidelines (average SBP ≥140 mm Hg or average DBP ≥90 mm Hg) to designate individuals with hypertension (HTN). With the proposed changes in these guidelines, it is expected that the prevalence of HTN would increase by 14%.

## Review

Well-elaborated management guidelines

HTN is classically managed with a plethora of interventions, ranging from pharmacological to non-pharmacological measures to decrease or manage increased BP. According to the 2017 Hypertension Clinical Guidelines by AHA, decisions to manage HTN using BP-lowering medications, in addition to non-pharmacological interventions, should be determined by the level of BP and the patient's risk for atherosclerotic cardiovascular disease (ASCVD) [2]. The most important early interventions are stated to be weight loss, the Dietary Approaches to Stop Hypertension (DASH) [3] diet, sodium reduction, potassium supplementation, increased physical activity, and reduction in alcohol consumption. Moreover, the guidelines also provided condition-related pharmacological interventions on the treatment of HTN.

Thiazide diuretics, calcium channel blockers (CCBs), angiotensin receptor blockers (ARBs), or angiotensin-converting enzyme (ACE) inhibitors [4] have been recommended as the first-line agents [5] for the initiation of pharmacological therapy in a newly diagnosed patient. Two first-line agents of different classes are recommended in adults with stage 2 HTN, and for stage 1 HTN, a single first-line agent is warranted with an active dose adjustment and additional agents according to the BP.

Hypertension with comorbidities

In addition, the guidelines provide varied treatment strategies for individuals diagnosed with HTN and other clinical comorbidities. Many pathologies affect clinical decision-making [6] in HTN. These include ischemic heart disease (IHD), heart failure with reduced ejection fraction (HFrEF), chronic kidney disease (CKD) (including renal transplantation), cerebrovascular disease, atrial fibrillation (AF), peripheral arterial disease (PAD), diabetes mellitus (DM), and metabolic syndrome. We illustrate some of the specific treatment guidelines for patients with concomitant disorders other than HTN. Certain risk factors [7] are also to be considered in hypertension, which aids in screening high-risk groups and suggesting appropriate interventions aiding early diagnosis and management.

Heart Failure with Reduced Ejection Fraction (HFrEF)

According to these guidelines, the recommended goal for adults with HFrEF and HTN is set at 130/80 mmHg [8-9]. Guideline Determined Medical Therapy (GDMT) beta blockers that include carvedilol, metoprolol succinate, and bisoprolol are the recommended first-line agents for these patients. Calcium channel blockers (CCBs) are not recommended in this scenario. Medications with compelling indications for HFrEF that may be used as first-line therapy to treat high BP include ARBs or ACE inhibitors, mineralocorticoid receptor antagonists, diuretics, and GDMT beta-blockers [3]. 

Chronic Kidney Disease (CKD)

HTN is the most prevalent clinical symptom associated with chronic kidney disease CKD [10]. About 67%-92% of patients with CKD have HTN [11]. A treatment course can reduce intraglomerular pressure and thereby reduce albuminuria [12], and serum creatinine may increase up to 30% because of the concurrent reduction in glomerular filtration rate (GFR) [4]. Patients with CKD also have a target goal of 130/80 mmHg. An ACE inhibitor (or an ARB, in the case of ACE inhibitor intolerance) is the most preferred treatment for patients suffering from HTN and CKD [13]. The use of ACE inhibitors has been recommended for all patients with stage 3 CKD or higher. ACE inhibitors or ARBs are also suggested for patients with stage 1 or stage 2 CKD with significant albuminuria, which is defined as -≥300 mg/d or ≥300 mg/g of albumin-creatinine ratio or equivalent in the first void in the morning. There is, in fact, a significant decrease in HTN without a loss in GFR with treatment on enalapril even if taken for prolonged periods of time [14]. After renal transplantation, patients suffer from HTN as a result of pre-existing kidney disease, the effects of immunosuppressive medications, and/or the presence of an allograft pathology [15]. Some percentage of post-transplant recipient patients develops donor-associated hypertension [16]. Patients treated with calcineurin inhibitor-based immunosuppression regimens have an HTN prevalence of 70% to 90% [17-18]. On the basis of the Cochrane analysis [19], treatment with CCBs proves to be the best, as they prevent graft loss and maintain a higher glomerular filtration rate (GFR) [5]. Patients with CKD and resistant hypertension are found to have salt-sensitive hypertension and the primary approach can be with simple salt restriction followed by aggressive pharmacotherapy [20].

Intracranial Hemorrhage

Elevated BP is highly prevalent in the setting of acute intracranial hemorrhage (ICH) [21]. It is linked to hematoma expansion, neurological worsening, death, and dependency after ICH. An immediate lowering of SBP to less than 140 mmHg is not advised in adults who present within six hours of the event with an SBP of 150-220 mmHg [22]. The use of intravenous anti-hypertensive agents has been recommended for all ICH patients with an SBP of 220 mmHg or higher. For acute ischemic stroke patients, the advantage of lowering BP early in reducing death and dependency is uncertain. Restarting antihypertensive therapy to improve long-term BP control is reasonable in these patients after the first 24 hours if they have pre-existing HTN and are neurologically stable.

Peripheral Arterial Disease

HTN is a common factor for patients with peripheral arterial disease (PAD) [23-24]. These patients are included in various clinical trials of antihypertensive drug therapy. However, the outcomes of various randomized controlled trials (RCTs) suggest that no superior drug has been noted for the treatment of HTN in patients with PAD. So, it has been recommended that HTN is treated in patients with concomitant PAD similarly to HTN patients with no PAD [25].

Atrial Fibrillation

Patients with atrial fibrillation (AF) have HTN in 80% of the cases [26] and it's the most common condition, regardless of age [27]. HTN has long been recognized as a risk factor for AF [28] because it is associated with left ventricular hypertrophy, deranged diastolic function with impaired left ventricular filling, raised left atrial pressures with left atrial hypertrophy and enlargement, increased atrial fibrosis, and slowing of intra-atrial and inter-atrial electrical conduction velocities. In addition, five randomized controlled trials RCTs were conducted to determine the best medication therapy and the results showed a superiority of renin-angiotensin system (RAS) blockade over CCBs [6,29]. Thus, ARBs are advised to prevent the recurrence of AF in HTN patients [30].

Diabetes Mellitus

The prevalence of HTN among adults with diabetes mellitus (DM) is approximately 80% [31], and HTN is at least twice as common in individuals with type 2 DM than in age-matched individuals without DM. In patients with DM, a threshold of 130/80 mmHg is set for the initiation of antihypertensive therapy. All first-line agents have been deemed equally effective in patients with DM and HTN. Various controlled trials and meta-analyses showed that ACE inhibitors and ARBs have the best efficacy among the HTN drug classes on urinary albumin excretion [32]. According to one meta-analysis of RCTs, ACE inhibitors or ARBs prevent moderate to severe albuminuria in patients with DM. According to these guidelines, in the presence of albuminuria, ACE inhibitors and ARBs might be used for patients with DM and HTN.

Follow-up

Treatment monitoring and the follow-up routine were also amended in the new guidelines. Substantial changes to various stages of HTN were included. For patients with normal BP, the guidelines suggest a yearly follow-up assessment. For patients with an elevated BP and stage 1 HTN, assessment should be made every three to six months with an assessment and optimized adherence therapy. Follow-up assessment every month is advised for patients with stage 2 HTN.

## Conclusions

Improving treatment and blood pressure (BP) control in adults with hypertension (HTN) involves a system of techniques and strategies that are individualized according to the needs of the patient. Hypertension Clinical Guidelines 2017 provides us with a well-researched tool that effectively improves current HTN management. It is a systematic algorithm for healthcare professionals who follow evidence-based clinical practice to restrain the complications brought by elevated BP.
